# Authors' Reply

**DOI:** 10.1371/journal.pmed.0030109

**Published:** 2006-02-28

**Authors:** Randall S Stafford, Veronica Monti, Jun Ma

**Affiliations:** **1**Stanford University Medical SchoolStanford, CaliforniaUnited States of America

Brown correctly points out that the rates of prophylactic aspirin use from our study [ 1] were much lower than the rates reported in some other United States studies, particularly those of Brown et al. [ 2] and O'Connor et al [ 3]. The latter studies represent practices in two large, integrated health maintenance organizations, which may represent special examples of best practices. Based on two nationally representative ambulatory-care surveys, our results are comparable to those from the Third National Health and Nutrition Examination Survey (NHANES III) [ 4]. As detailed in our discussion of data limitations, National Ambulatory Medical Care Survey and National Hospital Ambulatory Medical Care Survey data are reported by physicians on a per-patient visit basis, which may generate different findings than a population-based survey. Complementing earlier studies, our findings suggest that underutilization of aspirin as a cost-effective cardiovascular prevention strategy remains widespread in the US.

It is critical to discern factors associated with variation in aspirin use. This can lead to targeting some subpopulations for improvement or, alternatively, for attempts to promulgate features of best practice to other settings. For example, our analysis suggested that after adjusting for level of cardiovascular risk, women had significantly lower use of aspirin than men. As Brown points out, nonprofit-integrated medical care exemplifies one system in which prophylactic use of aspirin can be aggressively and effectively implemented. These settings have mechanisms in place that encourage sustained aspirin therapy, including guidelines, messages to clinicians, nursing-care management, alerts and routines embedded in electronic medical records, and direct mailings to patients [ 2]. Greater adoption of these mechanisms outside of integrated systems may have a favorable impact on national patterns of aspirin use. The issue of making our nation's health-care system more responsive to available evidence is a complex undertaking. We agree with Brown that direct-to-consumer drug advertising is a secondary factor in current patterns of aspirin use. Nonetheless, among other factors, direct-to-consumer drug advertising should be included as a potential barrier that may interfere with the translation of clinical evidence into practice.
